# Oxidative Stress and the NLRP3 Inflammasome: Focus on Female Fertility and Reproductive Health

**DOI:** 10.3390/cells14010036

**Published:** 2025-01-02

**Authors:** Efthalia Moustakli, Sofoklis Stavros, Periklis Katopodis, Charikleia Skentou, Anastasios Potiris, Periklis Panagopoulos, Ekaterini Domali, Ioannis Arkoulis, Theodoros Karampitsakos, Eleftheria Sarafi, Theologos M. Michaelidis, Athanasios Zachariou, Athanasios Zikopoulos

**Affiliations:** 1 Laboratory of Medical Genetics, Faculty of Medicine, School of Health Sciences, University of Ioannina, 45110 Ioannina, Greece; katopodisper@gmail.com; 2 Third Department of Obstetrics and Gynecology, University General Hospital “ATTIKON”, Medical School, National and Kapodistrian University of Athens, 12462 Athens, Greece; sfstavrou@med.uoa.gr (S.S.); apotiris@med.uoa.gr (A.P.); perpanag@med.uoa.gr (P.P.); garkoylis@hotmail.com (I.A.); theokarampitsakos@hotmail.com (T.K.); thanzik92@gmail.com (A.Z.); 3 Department of Obstetrics and Gynecology, Medical School of Ioannina, University General Hospital, 45110 Ioannina, Greece; haraskentou@gmail.com; 4 First Department of Obstetrics and Gynecology, Alexandra Hospital, Medical School, National and Kapodistrian University of Athens, 11528 Athens, Greece; kdomali@yahoo.fr; 5 Department of Biological Applications & Technology, School of Health Sciences, University of Ioannina, 45110 Ioannina, Greece; sarafi.elef@gmail.com (E.S.); tmichael@uoi.gr (T.M.M.); 6 Department of Urology, School of Medicine, University of Ioannina, 45110 Ioannina, Greece; zahariou@otenet.gr

**Keywords:** inflammation pathways, fertilization potential, redox balance, ovarian aging, mitochondria dysfunction, cytokine signaling

## Abstract

Chronic inflammation is increasingly recognized as a critical factor in female reproductive health; influencing natural conception and the outcomes of assisted reproductive technologies such as in vitro fertilization (IVF). An essential component of innate immunity, the NLR family pyrin domain-containing 3 (NLRP3) inflammasome is one of the major mediators of inflammatory responses, and its activation is closely linked to oxidative stress. This interaction contributes to a decline in oocyte quality, reduced fertilization potential, and impaired embryo development. In the ovarian milieu, oxidative stress and NLRP3 inflammasome activation interact intricately, and their combined effects on oocyte competence and reproductive outcomes are significant. The aims of this review are to examine these molecular mechanisms and to explore therapeutic strategies targeting oxidative stress and NLRP3 inflammasome activity, with the goal of enhancing female fertility and improving clinical outcomes in reproductive health.

## 1. Introduction

Female fertility is a complicated biological process that is controlled by the ovarian microenvironment, hormones, and cellular communication. A number of important activities, including follicle formation, oocyte maturation, and conception, depend on the ovarian microenvironment [1]. During the ovulation and luteinization stages, stress responses and cellular signaling must be properly coordinated since a balance between these elements is necessary for the best possible reproductive health [2].

Conversely, fertility may be influenced by illnesses that upset this equilibrium. Worldwide, 10–15% of couples experience infertility, with female infertility accounting for about 50% of instances. Polycystic ovarian syndrome (PCOS), endometriosis, age-related infertility, and infertility that cannot be explained are the main causes [3].

According to new research, oxidative stress and peripheral inflammatory responses may play a role in the emergence of these reproductive diseases and the ensuing infertility in women. More medical studies are necessary since the pathobiology of these processes is intricate and linked [4]. Fertility is greatly impacted by inflammation, a physiological process necessary for ovulation, implantation, and reproduction. For instance, a conditioned inflammatory response promotes cell division and muscle regeneration in embryos. However, there is a clear correlation between uterine malfunction and infertility and chronic low-grade inflammation, which is characterized by persistent immunological activation [5]. Premature ovarian insufficiency (POI), endometriosis, and PCOS are among the conditions that showcase the negative consequences of persistent inflammation [6].

Systemic inflammation in PCOS inhibits folliculogenesis and ovulation by exacerbating insulin resistance and hyperandrogenism. Ovarian reserves are depleted and ovarian aging is accelerated by autoimmune mechanisms and increased pro-inflammatory cytokines in POI, whereas chronic inflammatory lesions linked to endometriosis impair uterine function [7]. Oocyte quality, fertility, and the results of in vitro fertilization (IVF) and other assisted reproductive technologies (ART) are all adversely impacted by these inflammatory alterations. Targeted treatments are necessary to enhance female reproductive health and ART success rates since chronic inflammation and infertility are intertwined [8,9].

One important regulator of inflammation and innate immune responses is the pyrin domain-containing 3 (NLRP3) inflammasome, which belongs to the NLR family. Pro-inflammatory cytokines including interleukin-18 (IL-18) and interleukin-1β (IL-1β) are produced and released when caspase-1 activates this multiprotein complex [10,11]. Although it is useful for identifying tissue injury or inflammation, the NLRP3 inflammasome is also linked to persistent inflammation in the reproductive system.

The activation of the NLRP3 inflammasome is primarily triggered by oxidative stress. An imbalance between the generation of reactive oxygen species (ROS) and the ability of antioxidant mechanisms to neutralize them is the cause of this disorder [12]. ROS serve as signaling molecules in the ovarian microenvironment that promote oocyte maturation during follicular development and ovulation. DNA fragmentation, mitochondrial malfunction, and cellular damage are the results of high ROS generation [13].

Various variables, including age, environmental pollutants, and metabolic changes, can exacerbate these negative effects. In addition to its direct effect on oocyte quality, oxidative stress has been demonstrated to trigger an inflammatory cascade that lowers egg viability and impairs ovarian function by activating the NLRP3 inflammasome in granulosa and theca cells. Poor results may result from this feedback loop between oxidative stress and activation of the inflammasome during IVF [14,15].

This review highlights the combined effects of oxidative stress and NLRP3 inflammasome activation on female fertility by offering a thorough summary of recent studies in this area. To improve women’s reproductive health and optimize perinatal outcomes, it is important to highlight the possibility of new treatment targets that could lessen these problems.

## 2. Oxidative Stress and Female Reproductive Health

### 2.1. Definition and Biological Mechanisms of Oxidative Stress

The body experiences oxidative stress when the generation of ROS surpasses the antioxidant defenses’ ability to neutralize them. The majority of ROS are produced naturally as metabolic byproducts by the mitochondria’s oxidative phosphorylation mechanism. These comprise free radicals such as superoxide ions (O_2_^−^) and hydroxyl radicals (OH·), as well as non-radical species such as hydrogen peroxide (H_2_O_2_) [16].

Oxidative stress refers to an imbalance between the production of ROS and the body’s ability to neutralize them with antioxidants, which can lead to cellular damage and contribute to a variety of diseases, including those affecting the reproductive system. Several important biomarkers that can have an adverse influence on fertility and reproductive outcomes are linked to oxidative stress in the context of female reproductive health. A crucial indicator is nitrosative stress, which is brought on by the generation of reactive nitrogen species (RNS). The signaling molecule nitric oxide (NO) can cause RNS, particularly peroxynitrite, which can harm cellular components and obstruct reproductive functions. It has been demonstrated that excessive NO radicals degrade egg quality, hinder ovarian function, and have a detrimental effect on fertilization and embryo development [17].

Lipid peroxidation is another crucial indicator of oxidative stress that occurs when ROS target the polyunsaturated fatty acids in cellular membranes, producing compounds such as malondialdehyde (MDA) and 4-hydroxy-2-nonenal (HNE). These lipid peroxidation byproducts have the ability to negatively impact biological components, including oocyte membranes, which lowers the viability and fertilization potential of the cells [18]. Overall fertility, embryo development, and oocyte quality have all been found to decline with lipid peroxidation. Similarly, the oxidation of proteins is another hallmark of oxidative stress. Vital biological functions may be disrupted by oxidized proteins, which arise from ROS attacking cellular proteins and causing structural and functional alterations. Infertility can be caused by oxidized proteins in the reproductive system, which can disrupt hormone communication, change the maturation of eggs, and obstruct the implantation of embryos [4].

Antioxidant enzymes including glutathione peroxidase (GPx), catalase (CAT), and superoxide dismutase (SOD) are essential to the body’s natural defense against oxidative stress. By neutralizing ROS, these enzymes preserve homeostasis and guard against cellular harm. However, oxidative stress can overwhelm the reproductive system and cause malfunction if ROS levels are too high or antioxidant defense is insufficient [19]. Oxidative stress places granulosa cells and oocytes in the follicular milieu at particular risk. Their low ability to repair DNA and high metabolic activity make them especially vulnerable to damage by ROS. Their general reproductive health, as well as their function, is compromised by this oxidative damage [20] (Table 1). Fertility and pregnancy outcomes can be significantly impacted by a decrease in antioxidant enzyme activity or an excess of ROS. 

### 2.2. Effects of Oxidative Stress on Fertility

Oxidative stress has a major impact on many areas of female reproductive health. Oocyte quality is one crucial region impacted because oxidative damage hinders both nuclear and cytoplasmic maturation. Damage from ROS can cause double-strand breaks and point mutations in DNA, which lowers the chance of pregnancy [4]. Oocyte metabolism and developmental potential are further jeopardized by mitochondrial dysfunction, which is typified by reduced ATP production and mitochondrial membrane potential.

Additionally, follicular fluid, which is essential for promoting oocyte development and maturation, changes in composition due to oxidative stress [19]. Poorer oocyte retrieval during IVF cycles, higher lipid peroxidation, and decreased antioxidant capacity are all linked to elevated ROS levels in this microenvironment. Granulosa cells, which are crucial for oocyte development, are especially vulnerable to oxidative stress [20,21]. Important growth factors that are essential for follicular development, such as vascular endothelial growth factor (VEGF) and insulin-like growth factor (IGF-1), are not secreted by dysfunctional granulosa cells [22].

Oxidative stress interferes with implantation and early embryonic development in addition to oocyte maturation. Damaged gametes produce zygotes with poor developmental potential, and ROS-induced inflammation in the uterine lining lowers implantation success rates. Oxidative stress also contributes significantly to ovarian aging and premature ovarian insufficiency (POI) [4,17]. Chronic oxidative stress speeds up ovarian aging by causing oocytes to undergo apoptosis and decreasing the follicular pool. Ovarian reserve decreases early in POI due to oxidative stress, which is frequently associated with autoimmune and mitochondrial abnormalities [13,14] (Table 2).

ROS may impair cells, resulting in issues such as inadequate follicular fluid, decreased egg quality, impaired cell function, and difficulty with embryo implantation. Primary ovarian insufficiency (POI) and other disorders can be exacerbated by oxidative stress, which can also accelerate ovarian aging. Inflammation, issues with cell energy production, and DNA damage are the main causes of these effects.

### 2.3. Clinical Proof of the Association Between Female Infertility and Oxidative Stress

Numerous studies have demonstrated the negative impact of oxidative stress on female fertility. In women undergoing in vitro fertilization (IVF), lower rates of oocyte retrieval, fertilization, and implantation are linked to elevated levels of oxidative stress indicators in serum or follicular fluid. Furthermore, high concentrations of NO radicals have been linked to impaired ovarian function, reduced egg quality, and poor fertility outcomes. In patients with polycystic ovarian syndrome (PCOS), elevated ROS are also associated with insulin resistance, hyperandrogenism, and persistent anovulation, all of which have a detrimental effect on fertility [23]. Similarly, in endometriosis patients, decreased implantation success rates and inferior oocyte quality are associated with higher levels of oxidative stress in the peritoneal fluid. Clinical assessments of oxidative stress markers, including lipid peroxidation levels and antioxidant enzyme activities, are increasingly used as diagnostic tools to evaluate reproductive health. These biomarkers are valuable in understanding the underlying causes of infertility and may provide insights into potential therapeutic interventions. According to these results, oxidative stress actively contributes to the pathophysiology of infertility rather than just acting as a passive spectator [21,24].

## 3. The NLRP3 Inflammasome in Reproductive Health

### 3.1. Overview of the NLRP3 Inflammasome

The NLRP3 inflammasome is one protein that belongs to the Nod-like receptor (NLR) family. It is vital for the innate immune response because it is involved in reacting to damage-associated molecular patterns (DAMPs) or pathogen-associated molecular patterns (PAMPs) under cellular stress [25].

Several crucial procedures are involved in the assembly of the NLRP3 inflammasome. As a sensor in this process, activation of the NLRP3 protein encourages the creation of inflammasomes. NLRP3 is connected to downstream signaling molecules by the adaptor protein ASC (apoptosis-associated speck-like protein containing a CARD) [11]. One important effector, caspase-1, promotes the proteolytic activation of pro-inflammatory cytokines such as interleukin-1β (IL-1β) and interleukin-18 (IL-18), and it causes pyroptosis, a type of programmed cell death [26]. The NLRP3 inflammasome increases the inflammatory response by promoting the maturation of IL-1β and IL-18. Chronic activation or dysregulation of the inflammasome can result in disorders linked to inflammation, including those that affect reproductive health. However, since it encourages tissue healing and offers infection prevention, regulated activation is beneficial [27].

### 3.2. Mechanisms of NLRP3 Inflammasome Activation

The NLRP3 inflammasome is activated by a variety of signals that fall into a few broad categories. Bacterial toxins, viral RNA, and lipopolysaccharides (LPS) are examples of microbial stimuli that activate Toll-like receptor (TLR)-mediated pathways. Inflammasomes may be activated by increasing advanced glycation end products (AGEs), cholesterol crystals, and excessive glucose levels, all of which are factors in metabolic stress [11,28].

Oxidative stress, which is defined by an excess of ROS, mitochondrial damage, and disruptions in cellular homeostasis, directly triggers the NLRP3 inflammasome. Moreover, endocrine disruptors and other environmental contaminants are linked to elevated inflammasome activity [29]. Prior to NLRP3 inflammasome activation, critical events include lysosomal instability, mitochondrial malfunction, and ionic imbalances. NLRP3 inflammasome activation has been thought to be frequently triggered by a decrease in intracellular K^+^. Furthermore, K^+^ efflux is not necessary for the alternative NLRP3 inflammasome pathway, but it is necessary for NLRP3 activation in the caspsase-11-mediated non-canonical inflammasome pathway. Additionally, 1L-1β maturation is promoted by the activation of Ca^2+^-independent phospholipase A2 brought on by potassium efflux [11]. Heavy metals and air pollution play a major role in inflammasome activation in the oocyte microenvironment. These factors often coexist with oxidative stress, perpetuating the inflammatory cycle [30] (Table 3).

NLRP3 is activated by microbial stimuli, such as viruses and bacteria, via immunological pathways. Environmental contaminants, metabolic stress, and oxidative stress from excessive ROS and mitochondrial damage all activate NLRP3 and are associated with increased inflammasome activity.

### 3.3. Expression and Role of the NLRP3 Inflammasome in the Reproductive System

There is growing recognition of the role of the NLRP3 inflammasome in controlling female fertility and ovarian function. The NLRP3 inflammasome is expressed by important ovarian cell types, such as granulosa cells, theca cells, and oocytes, which cause inflammatory reactions in both healthy and diseased circumstances [12].

Normal reproductive functions, including uterine remodeling, cytokine release, and local immune cell activation, depend on the NLRP3 inflammasome. A viral outbreak-like phenomenon is frequently used to describe its activation. The NLRP3 inflammasome in granulosa cells temporarily activates cytokines such as interleukin-1β (IL-1β), which are necessary for follicle rupture and oocyte release [25].

Numerous congenital conditions have been connected to inflammasome malfunction at infection sites. Chronic activation in polycystic ovarian syndrome (PCOS) is linked to increased granulosa cell production of NLRP3 inflammasome components. In addition to impairing follicle formation and ovarian maturation, this aberrant inflammasome activation in ovarian and endometrial tissue exacerbates hyperandrogenism. In addition, it results in inflammation and oxidative damage to the endometrium, which lowers oocyte quality and reserves [31].

Additionally, in primary ovarian insufficiency (POI), chronic activation of the NLRP3 inflammasome speeds up follicular atresia and apoptosis, resulting in early ovarian aging. According to these findings, the NLRP3 inflammasome may be a novel therapeutic target for the management of infertility and may be linked to the age-dependent decline in female fertility [32].

### 3.4. Interplay Between the NLRP3 Inflammasome and Expression of Oxidative Stress and the Role of the NLRP3 Inflammasome in the Reproductive System

Activation of the NLRP3 inflammasome and oxidative stress are closely related feedback loops that worsen ovarian inflammation. NLRP3 is a major factor in the buildup of inflammasomes because ROS harm mitochondria and upset the ionic balance in cells. ROS quickly expose inflammasome components by activating upstream pathways such as NF-κB [11].

Once engaged, the NLRP3 inflammasome increases ROS production, damages mitochondrial function, and erodes the immune system. The structure and function of the placenta and its supporting cells are compromised by this exposure cycle, which exacerbates uterine inflammation [33]. In circumstances such as in vitro fertilization (IVF), when ovarian hormone suppression raises oxidative stress levels, this interaction is especially important [34] (Table 4).

Lower ovarian reserves result from the disruption of follicular development. Prolonged inflammation can damage the uterus and oocyte quality, which can impair embryo development and implantation.

### 3.5. Therapeutic Targeting of the NLRP3 Inflammasome 

The important function of the NLRP3 inflammasome in reproductive disorders makes it a promising therapeutic target to increase female fertility. Potential intervention techniques include cytokine blocking, lifestyle changes, anti-inflammatory medications, and inflammasome inhibitors [32]. Due to their encouraging results in preclinical investigations of inflammatory illnesses, small compounds such as MCC950, which selectively suppress NLRP3 activation, are being studied for their potential applications in reproductive health [35]. It has been determined that NLRP3 interacts with melatonin, CoQ10, and N-acetylcysteine (NAC).

Antioxidants assist in lowering oxidative stress by blocking these interactions [36]. Furthermore, anti-IL-1β and anti-IL-18 antibodies may lessen the negative effects on inflammasome function, which could enhance the results of assisted reproductive technologies such as IVF and restore ovarian function. Inflammasome overactivity can be decreased by lowering oxidative stress and systemic inflammation by lifestyle modifications such as regular exercise, a nutritious diet, and quitting smoking [37].

## 4. Intercoupling of Oxidative Stress and the NLRP3 Inflammasome

The oocyte microenvironment is greatly impacted by the intricate and reciprocal interaction that exists between oxidative stress and the NLRP3 inflammasome [36]. NLRP3 inflammasome activation is largely triggered by oxidative stress, and inflammasome activity also affects mitochondrial function. Through the increased generation of ROS, this cyclic interaction raises oxidative stress, encourages chronic inflammation, and causes cell malfunction, which ultimately results in issues with reproductive health [36,38].

Excess ROS produced by oxidative stress triggers the NLRP3 inflammasome, leading to ionic imbalances, lysosomal instability, and mitochondrial damage. The inflammasome interprets these situations as warning signs of danger. NLRP3, pro-caspase-1, pro-IL-1β, and pro-IL-18 are among the inflammasome components that are expressed as a result of ROS-stimulating redox-sensitive pathways such as NF-κB [12]. Oxidative stress is a key trigger in the activation of the inflammasome, and antioxidants have been demonstrated to prevent this process by lowering ROS levels.

Conversely, NLRP3 inflammasome activation on its own causes mitochondrial malfunction, which raises ROS production even more and prolongs oxidative stress [38]. Pyroptosis is a type of programmed cell death brought on by caspase-1 activity and inflammasome accumulation. ATP and mitochondrial DNA are two more danger signals produced by this process, which worsen oxidative stress and inflammation in nearby cells [39]. This interaction is especially harmful in the context of oocytes, where inflammatory and redox activities are vital and need to be balanced [40].

NLRP3 inflammasome activation and oxidative stress are coupled through a number of overlapping molecular pathways. A major contributing factor is mitochondrial malfunction, since damaged mitochondria emit metabolites such as ROS, mitochondrial DNA, and cardiolipin [41]. The NLRP3 inflammasome is directly stimulated by these compounds. Mitochondrial damage is therefore further exacerbated by decreased oxidative phosphorylation and elevated mitochondrial permeability [42].

This connection is also mediated by redox-sensitive pathways, specifically the NF-κB signaling cascade. Increased ROS levels cause IκB kinase to become active, which phosphorylates and degrades IκB and encourages NF-κB nuclear translocation [43]. The transcription of genes linked to inflammasomes is improved by this process. Important triggers for NLRP3 activation also include oxidative stress-induced disruptions in ion flux, including potassium efflux, calcium influx, and sodium imbalance. Ion channels are immediately impacted by ROS, which intensifies these ion imbalances [44]. Under oxidative stress, thioredoxin-interacting protein (TXNIP) separates from thioredoxin, forming another important mechanism. This connection is further enhanced by the binding and activation of NLRP3 by free TXNIP [45].

These closely related processes show how vulnerable the oxidative stress–NLRP3 inflammasome axis is, especially in the ovarian microenvironment, where disturbed inflammatory and redox balance might have serious consequences for the reproductive health of females [13].

### 4.1. Role of the Oxidative Stress–NLRP3 Axis in Ovarian Dysfunction

The interaction between oxidative stress and NLRP3 inflammasome activation has significant pathogenic implications in various ovarian diseases [46]. In polycystic ovary syndrome (PCOS), elevated levels of ROS in ovarian tissue and increased expression of NLRP3 components lead to chronic low-grade inflammation. This inflammation contributes to reproductive complications in women with PCOS, as it inhibits oocyte maturation and steroid hormone production [47]. In endometriosis, increased oxidative stress and NLRP3 inflammasome activation aggravate inflammatory lesions, resulting in tissue damage, adhesions, and infertility [48].

Follicle apoptosis is increased by oxidative stress and inflammasome activation, which has an effect on fetal aging as well [14]. Mitochondrial dysfunction is especially risky for older women since it exacerbates oxidative damage and inflammasome activation [49]. Ultimately, oxidative stress and NLRP3 activation have a significant impact on the results of in vitro fertilization (IVF), since they are associated with poor oocyte quality, decreased pregnancy success, and delayed embryonic development [50] (Table 5).

In polycystic ovarian syndrome, they lead to oocyte maturation impairment, hormonal imbalance, and inflammation. Oxidative damage speeds up cell death and decreases ovarian follicles in age-related ovarian aging.

### 4.2. Therapeutic Implications in Targeting the Oxidative Stress–NLRP3 Axis

Reproductive results may be enhanced by addressing the connection between oxidative stress and the NLRP3 inflammasome. Antioxidants, lifestyle changes, combination therapy, and direct inflammasome inhibitors are examples of potential treatment approaches [51].

Antioxidants are at the forefront of such strategies, offering dual benefits of reducing oxidative damage and mitigating inflammasome activation. Melatonin, a powerful endogenous antioxidant, stabilizes mitochondrial function, reduces ROS generation, and directly inhibits NLRP3 activation. Melatonin reduces ROS formation and inhibits NLRP3 activation during IVF cycles, improving the quality of the oocytes. It stabilizes the mitochondria and has strong antioxidant properties [52]. Clinical studies indicate that melatonin supplementation during IVF cycles enhances oocyte quality, fertilization rates, and embryo development. Similarly, coenzyme Q10 (CoQ10) enhances mitochondrial efficiency, lowers oxidative damage, and indirectly attenuates NLRP3 activity.

N-acetylcysteine (NAC), a precursor of glutathione, further strengthens cellular defenses against ROS while reducing inflammatory cytokines within the follicular fluid. A healthier ovarian microenvironment is promoted by this dual effect, which raises IVF success rates overall and improves oocyte quality. In addition to their beneficial impact on ROS, vitamins C and E also prevent oxidative damage to biological components, opening up new treatment options.

As promising treatments for reproductive health, direct inflammasome inhibitors such as MCC950 have demonstrated effectiveness in preclinical models of inflammation by directly blocking NLRP3 activation [53]. Changes in lifestyle, such as consistent exercise, antioxidant-rich foods, and stress reduction, can lower inflammation and systemic oxidative stress, which in turn lowers NLRP3 activation and supports reproductive health [54].

Combination treatments that simultaneously target oxidative stress and inflammatory pathways may have further advantages. Given that both systems are less effective in treating diseases such as endometriosis and PCOS, this method may be very useful in treating these conditions and offering a holistic therapeutic approach [5,55].

## 5. Impact on In Vitro Fertilization (IVF) Outcomes

### 5.1. Overview of IVF and the Role of the Ovarian Microenvironment

IVF is a crucial component of assisted reproductive technology (ART) that involves controlled sperm stimulation, egg harvesting, insemination, and embryo transfer. However, a number of obstacles, such as problems with oocyte quality, oocyte retrieval, and embryo development, restrict the success rates [4,21,50].

The oocyte microenvironment has a significant impact on the quality of oocytes recovered during an IVF cycle. A delicate balance between inflammation and oxidative stress is necessary for the best follicle growth and oocyte maturation. IVF results can be greatly impacted by dysregulation of this balance, which is typified by elevated oxidative stress and persistent inflammation brought on by NLRP3 inflammasome activation [21].

Controlled ovarian hyperstimulation (COH) is one of the IVF techniques that naturally increases oxidative stress in the oocyte microenvironment [21]. There are multiple reasons behind this rise in oxidative stress. Gonadotropin doses given during COH stimulate hormones, which causes the ovaries’ ROS levels to rise noticeably [4]. Oxidative stress is further exacerbated by the stimulated ovarian follicular fluid’s frequent high concentrations of ROS and inflammatory cytokines. Additionally, prolonged in vitro culture conditions might harm cells oxidatively, reducing their capacity to proliferate [50].

There are a number of adverse consequences linked to elevated oxidative stress during IVF cycles [21]. Lower oocyte quality results from ROS-induced damage to proteins, lipids, and mitochondrial DNA (mtDNA), which hinders oocyte development and survival. Pregnancy rates are decreased as a result of this oxidative damage, which also impacts the fragile cellular machinery required for effective reproduction. Furthermore, chromosomal abnormalities and significant developmental delays are more likely to occur in embryos formed from oxidatively stressed oocytes, which lowers the likelihood of a successful pregnancy and degrades the quality of the embryo overall [50].

### 5.2. NLRP3 Inflammasome and IVF Outcomes

Chronic NLRP3 inflammasome activation during the IVF cycle worsens inflammation and oxidative damage in the oocyte microenvironment, which can have negative effects. The reproductive system’s essential components are disturbed by this overactivation [21].

First, it has a detrimental effect on follicle growth and oocyte quality. Granulosa cells are unable to sustain healthy follicle growth when the inflammasome is overactivated. Interleukin-1β (IL-1β) and interleukin-18 (IL-18), two inflammatory cytokines, are released into the follicular fluid and are linked to low oocyte quality and a decreased rate of oocyte maturation [4,50]. Oocyte apoptosis is caused by prolonged inflammasome activation, which exacerbates this problem. It further reduces the capacity for oocyte development by inducing mitochondrial malfunction [50].

Additionally, there are negative impacts on the development of the embryo and pregnancy. Sperm abnormalities result from oocyte fertilization that fails to acquire appropriate developmental competence when regulated inflammasome activity is present. In addition to causing embryonic fragmentation and reducing blastocyst development, elevated levels of inflammatory cytokines and ROS in the follicular fluid also degrade sperm quality [56,57].

Lastly, there are significant effects on implantation and embryo retrieval. The uterus’s capacity to sustain implantation is compromised by inflammation mediated by inflammasomes, raising the possibility of implantation failure. Higher intraovarian IL-1β levels are significantly associated with lower implantation success during ART cycles, which further limits the total effectiveness of IVF procedures [21,50].

### 5.3. Clinical Data Connecting the NLRP3 Inflammasome and Oxidative Stress to IVF Results

Recent clinical research shows that oxidative stress and inflammasome activation have a major effect on IVF results. Poor oocyte and ovarian function are linked to elevated levels of ROS in the follicular fluid as well as pro-inflammatory cytokines such as interleukin-1β (IL-1β) and interleukin-18 (IL-18) [58]. The success of IVF may be predicted by these biomarkers. Additionally, granulosa cells from individuals with insufficient or recurrent ovarian reserve failure have been found to express higher NLRP3 inflammasome components, indicating a clear correlation between inflammasome activity and poor reproductive outcomes [59].

Age is also a significant factor since older IVF patients have greater levels of oxidative stress and inflammasome activation, which results in age-related declines in fertility and poorer ART success rates. These findings underscore the particular challenges faced by high-risk or elderly patient populations undergoing IVF [60]. This demonstrates how oxidative stress and inflammasome function can be monitored and altered as therapeutic approaches. IVF success rates may be increased by using elevated oxidative stress and inflammasome activity as indicators and therapeutic targets, allowing for more individualized and efficient care for these patients [50,61].

### 5.4. Therapeutic Approaches to Mitigate the Impact

Since oxidative stress and NLRP3 inflammasome activation have been demonstrated to adversely impact IVF outcomes, a number of treatments have been studied to mitigate these factors [12]. Antioxidants are a prominent approach to improving success rates. For instance, melatonin, known for its mitochondrial protective properties, has been demonstrated to enhance oocyte quality and lessen oxidative damage to follicular fluid during IVF cycles [62]. Similarly, coenzyme Q10 improves oocyte responsiveness and sperm morphology by enhancing mitochondrial function in mature oocytes. Vitamins E and C scavenge ROS within the oocyte microenvironment, preventing oxidative damage during follicle development [63].

NLRP3 inflammasome inhibitors are a novel and developing strategy. By directly suppressing the NLRP3 inflammasome, compounds such as MCC950 have demonstrated potential in preclinical models. However, their application in reproductive treatment is still being investigated. Because they can lower inflammasome activity, these substances may help with inflammatory IVF failures [64].

Changes in lifestyle can also have a significant impact on lowering systemic oxidative stress and enhancing reproductive results. Oxidative stress can be reduced and overall fertility can be improved by reducing stress, stopping smoking, and eating a diet high in antioxidants [65].

Optimizing IVF protocols during controlled ovarian hyperstimulation (COH) offers another avenue to address oxidative stress. Oxidative damage may be reduced by lowering gonadotropin dosages and customizing stimulation regimens for each patient [66]. Additionally, growth-promoting conditions, such as low-oxygen environments, can shield developing cells from oxidative stress [67].

Lastly, anti-inflammatory medications that target inflammation mediated by inflammasomes represent a complementary approach [68]. Patients with inflammation-related IVF difficulties may benefit from cytokine-neutralizing antibodies or IL-1β blockers, which may enhance embryo receptivity and boost fertilization success [69]. Both oxidative stress and inflammation are addressed by these systems, which combine to improve IVF results in a number of ways.

### 5.5. Future Directions in Research and Clinical Practice

Even though we now know more about the relationship between oxidative stress, NLRP3 inflammasome activation, and IVF outcomes, further study is still required to improve clinical applicability and success rates. The effects of oxidative stress on follicular fluid and granulosa cells, as well as the control of inflammasome activity, are of significant interest. To show their potential in lowering inflammasome-induced IVF failure, more research is required to assess the safety and effectiveness of inflammasome inhibitors in ART settings in addition to creating trustworthy biomarkers that may be used as indicators of IVF success [14].

Targeting the distinct oxidative and inflammatory profiles of each patient through customized treatment plans is another important field of research. These programs can enhance outcomes by customizing interventions to meet the needs of each individual. However, it is crucial to investigate the long-term impacts of these methods on pregnancy outcomes and the health of the children to guarantee that they are safe and effective in the larger context of reproductive and developmental health [70].

## 6. Therapeutic Interventions and Future Directions

### 6.1. NLRP3 Inflammasome Inhibitors

Targeting the NLRP3 inflammasome directly is one method that appears to have potential for lowering intrauterine inflammation and maybe improving IVF outcomes. One important strategy is the application of MCC950, a small-molecule inhibitor that preserves upstream priming pathways while specifically inhibiting the activity of the NLRP3 inflammasome [36]. Preclinical investigations have demonstrated that MCC950 effectively inhibits the release of interleukin-1β (IL-1β) and minimizes oxidative damage in inflammatory situations, making it a promising candidate for fertility treatment [71].

Another intriguing option is the use of IL-1β inhibitors, such as neutralizing antibodies and vaccinations. By activating IL-1β, the NLRP3 inflammasome causes inflammation. IL-1β inhibitors have the potential to improve uterine receptivity and decrease chronic inflammation in IVF patients, which would raise the likelihood of a successful implantation. These inhibitors have the potential to enhance IVF results, despite their primary research being in autoimmune illnesses [72].

Additionally, ROS-modulating therapies offer an indirect way to prevent the activation of the NLRP3 inflammasome. MitoQ and other mitochondria-targeted antioxidants lower ROS to help stop oxidative stress and the activation of the inflammasome. Combining these processes could lead to new developments in fertility treatments by preventing reproductive problems and improving assisted reproductive technology [36] (Table 6).

Consuming foods rich in polyphenols and antioxidants helps modulate NLRP3 and lower stress. Regular exercise enhances vitality and reduces inflammation, though excessive exercise may increase oxidative or physical stress.

### 6.2. Innovative Strategies and Future Directions in Reproductive Health

Promising opportunities to enhance IVF results and reproductive health are presented by novel treatments that target the oxidative stress–NLRP3 inflammasome axis [36]. NLRP3 or related inflammasome genes in reproductive organs can be silenced by gene therapy and RNA interference using instruments such as CRISPR-Cas9 [73]. Additionally, inflammasome components can be targeted in reproductive therapy using antisense oligonucleotides and small interfering RNAs (siRNAs), which have already been produced for other disorders [74]. Further gaining popularity is mitochondrial replacement treatment, which involves putting mitochondria into healthy embryos or oocytes. For patients with mitochondrial dysfunction, this strategy shows potential. More sophisticated treatments that target the mitochondria, including SS-31 peptides, are being researched to improve the quality of oocytes and embryos, lessen oxidative damage, and restore mitochondrial function [75].

Another developing subject is microbial regulation, since studies indicate that vaginal and gut bacteria affect inflammation and systemic oxidative stress. According to reproductive health, probiotics and prebiotics may offer novel ways to control the oxidative–inflammatory axis, making fertility higher. Antioxidants and ROS-scavenging substances are also being added to IVF culture media to lessen oxidative stress during in vitro embryo development. This will increase embryo viability, development efficiency, and IVF success rates [76].

Many questions still surround the oxidative stress–NLRP3 inflammasome axis in reproductive health, despite tremendous advancements in our understanding of this relationship. In follicular fluid, granulosa cells, and uterine tissue, future studies should concentrate on creating trustworthy indicators of oxidative stress and inflammasome activity [14,21,32].

Additionally, non-invasive indicators in blood or urine may improve the tracking of inflammatory and systemic oxidative diseases in IVF patients, allowing for more individualized therapy planning. Tailored treatment customized based on individual oxidative stress and inflammation profiles is key [77]. Machine learning algorithms and other AI-driven tools can evaluate these profiles to predict disease risk and enhance individualized treatment regimens, which helps to improve disease prevention and control. To assess oxidative stress levels, for instance, AI-based predictive models examine biomarkers, and machine learning tailors interventions by combining clinical and molecular data. By facilitating early diagnosis, customized therapy, and improved monitoring in reproductive health, these strategies enhance therapeutic outcomes [78]. More precise and successful interventions may result from the use of AI-driven analytics to integrate genetic, metabolic, and lifestyle aspects.

Examining these treatments’ long-term impacts is also crucial, especially the potential effects of oxidative stress and inflammasome modification during IVF on long-term health [79]. Lastly, the combination of transcriptomics, proteomics, metabolomics, and genomics in multi-omics techniques holds great promise for the discovery of new therapeutic targets. These methods increase our comprehension of fertility procedures and results, which eventually results in more potent therapies [80].

## 7. Conclusions

Oxidative stress and inflammation have a significant effect on female reproductive health, particularly when the NLRP3 inflammasome is activated. One aspect of ovarian activity is oxidative stress, which impairs embryonic growth, fertilization potential, and egg quality. Chronic activation of NLRP3 causes inflammation, which adversely affects the outcome of IVF and biological reproduction. Follicle production, oocyte maturation, and embryo implantation are all impacted by the vicious cycles of ovarian and uterine cell damage caused by oxidative stress and inflammasome activation. Since infertile patients often have elevated levels of oxidative stress markers and cytokines associated with the inflammasome, targeted treatments are crucial. Antioxidants that reduce oxidative damage, such as melatonin, coenzyme Q10, and N-acetylcysteine, can enhance IVF outcomes. Among the possible anti-inflammatory medications are interleukin-1β inhibitors and MCC950. Additionally crucial are lifestyle changes and tailored treatment plans based on each person’s particular oxidative and inflammatory profile. Further research is needed to identify biomarkers and develop targeted treatments. Microbiological research, drugs that target the mitochondria, and the development of gene therapy could all significantly improve reproductive results. Improving women’s fertility and IVF success requires addressing these variables, which will pave the way for more suitable and efficient reproductive care.

## Figures and Tables

**Table 1:** The table describes the mechanisms and effects on reproductive health of the extrinsic and intrinsic variables that lead to oxidative stress in the ovaries.

Factor	Description	Impact
Mitochondrial dysfunction	Ovarian cells’ mitochondria, including granulosa cells and oocytes, are the main generators of ROS. Dysfunction inhibits ATP synthesis and increases ROS generation.	Induces mitochondrial deficiencies, oxidative stress buildup, and decreased oocyte quality.
Environmental toxins	Pollutant exposure affects the redox balance of cells, including pesticides, heavy metals, and substances that disrupt hormones.	Reduces antioxidants or directly produces ROS, which increases oxidative stress in ovarian tissues and jeopardizes reproductive capacity.
Inflammatory cytokines	ROS generation is stimulated by cytokines such as TNF-α and IL-6, which are elevated in conditions such as PCOS and endometriosis.	Contributes to follicular dysfunction and decreased oocyte competence by raising oxidative stress in ovarian cells and follicular fluid.
Aging	Advanced maternal age increases the generation of ROS in oocytes while decreasing antioxidant efficiency (e.g., SOD and GPx).	Oocyte quality and ovarian reserves are decreased, which leads to infertility and poor ART results.
Lifestyle factors	High-fat diets, heavy alcohol use, and smoking all weaken antioxidant defenses or release exogenous ROS.	Increases oxidative stress and impairs ovarian tissues, especially in vulnerable ovarian cells.

**Table 2:** This table illustrates several facets of reproductive health that are impacted by oxidative stress.

Aspect of Reproductive Health	Impact of Oxidative Stress	Mechanism
Oocyte quality	Compromised nuclear and cytoplasmic maturation, leading to reduced fertilization potential.	DNA damage (double-strand breaks, mutations) and mitochondrial dysfunction (reduced ATP production and membrane potential).
Follicular fluid composition	Poorer oocyte retrieval during IVF cycles and reduced support for oocyte maturation.	Elevated ROS levels, reduced antioxidant capacity, and increased lipid peroxidation.
Granulosa cell function	Impaired secretion of growth factors critical for follicle development.	Damage to granulosa cells decreases VEGF and IGF-1 secretion, reducing metabolic and structural support for oocytes.
Implantation and embryonic development	Reduced chances of implantation and compromised embryonic potential.	Oxidative damage to gametes and ROS-mediated inflammation in the uterine lining impair zygote development and implantation success.
Ovarian Aging and POI	Accelerated ovarian aging, follicle pool depletion, and early ovarian reserve loss in POI.	Chronic oxidative damage promotes apoptosis in oocytes. Linked to autoimmunity and mitochondrial dysfunction in POI cases.

**Table 3:** This table summarizes the various stimuli that can cause the NLRP3 inflammasome to become active.

Stimuli	Description
Microbial stimuli	Lipopolysaccharides (LPS), bacterial toxins, and viral RNA activate NLRP3 through TLR-mediated pathways.
Metabolic stress	Elevated glucose, cholesterol crystals, and advanced glycation end products (AGEs) trigger inflammasome activation.
Oxidative stress	Excess ROS, mitochondrial damage, and disrupted cellular homeostasis directly activate NLRP3.
Environmental toxins	Heavy metals, air pollutants, and endocrine disruptors are linked to increased inflammasome activity

**Table 4:** Reproductive health is impacted by chronic NLRP3 activation, as this table illustrates.

Aspect of Reproductive Health	Impact of Chronic NLRP3 Activation
Oocyte Quality and Competence	Persistent inflammasome activation damages oocyte mitochondria, DNA integrity, and cytoplasmic maturation, leading to reduced fertilization potential. Elevated levels of IL-1β and IL-18 in follicular fluid have been correlated with lower IVF success rates in patients with poor ovarian response.
Follicular Development	Cytokine release mediated by the inflammasome disrupts granulosa cell function, impairing hormone synthesis and normal follicular development. Excessive inflammation accelerates follicular atresia and reduces ovarian reserve.
Embryo Development and Implantation	Oocytes from inflammasome-dysregulated environments tend to produce embryos with compromised developmental competence. Additionally, uterine inflammation driven by NLRP3 activity can hinder endometrial receptivity, lowering implantation rates in IVF.

**Table 5:** This table illustrates the effects of NLRP3 activation and oxidative stress on different reproductive situations.

Condition	Role of Oxidative Stress–NLRP3 Interplay
Polycystic Ovary Syndrome	Elevated ROS and NLRP3 activation lead to chronic inflammation, disrupted steroidogenesis, and impaired oocyte maturation.
Endometriosis	Heightened oxidative stress and NLRP3 activation perpetuate tissue damage, adhesions, and infertility.
Age-related ovarian aging	Accelerates apoptosis and depletion of ovarian follicles, with mitochondrial dysfunction worsening oxidative damage.
In Vitro Fertilization (IVF)	Increased oxidative stress and NLRP3 in follicular fluid correlate with poor oocyte quality and reduced embryo competence.

**Table 6:** This table illustrates how lifestyle changes can reduce oxidative stress.

Lifestyle Changes	Description
Dietary modifications	Systemic oxidative stress is reduced when antioxidant-rich foods, such as fruits, vegetables, nuts, and seeds, are consumed. NLRP3 activity is modulated by polyphenols such as curcumin and resveratrol.
Exercise	Inflammation is decreased and mitochondrial efficiency is increased with regular moderate exercise. Excessive activity can exacerbate oxidative stress, highlighting the need for balance.
Stress reduction	Inflammation and oxidative damage are exacerbated by ongoing psychological stress. Improved IVF results have been linked to practices such as yoga and mindfulness.

## Data Availability

Not applicable.
